# Depression and HIV Care-seeking Behaviors in a Population-based Sample in North West Province, South Africa

**DOI:** 10.1007/s10461-023-04102-3

**Published:** 2023-06-17

**Authors:** Lynae A. Darbes, Alison M. El Ayadi, Jennifer M. Gilvydis, Jessica Morris, Elsie Raphela, Evasen Naidoo, Jessica S. Grignon, Scott Barnhart, Sheri A. Lippman

**Affiliations:** 1 Department of Health Behavior and Biological Sciences, School of Nursing, University of Michigan, Ann Arbor, MI USA; 2 Department of Medicine, Division of Prevention Science, University of California, San Francisco, San Francisco, CA USA; 3 Department of Obstetrics and Gynecology, School of Medicine, University of California, San Francisco, San Francisco, CA USA; 4 Department of Global Health, Schools of Medicine and Public Health, University of Washington, Seattle, WA USA; 5 International Training and Education Center for Health (I-TECH), South Africa, Pretoria, Republic of South Africa; 6 Department of Medicine, Schools of Medicine and Public Health, University of Washington, Seattle, WA USA

**Keywords:** HIV, depression, HIV testing, ART adherence

## Abstract

Depression is associated with key HIV-related prevention and treatment behaviors in sub-Saharan Africa. We aimed to identify the association of depressive symptoms with HIV testing, linkage to care, and ART adherence among a representative sample of 18–49 year-olds in a high prevalence, rural area of South Africa. Utilizing logistic regression models (N = 1044), depressive symptoms were inversely associated with reported ever HIV testing (AOR 0.92, 95% CI 0.85–0.99; p = 0.04) and ART adherence (AOR 0.82, 95% CI: 0.73–0.91; p < 0.01) among women. For men, depressive symptoms were positively associated with linkage to care (AOR: 1.21, 95% CI: 1.09–1.34; p < 0.01). Depression may adversely impact ART adherence for HIV-positive women and reduce the likelihood of HIV testing for women not aware of their HIV status which, in settings with high HIV prevalence, carries severe consequences. For HIV-positive men, findings suggest that depression may encourage help-seeking behavior, thereby impacting their health system interactions. These findings underscore the need for health-care settings to factor mental health, such as depression, into their programs to address health-related outcomes, particularly for women.

## Resumen

La depresión está asociada con conductas clave de prevención y tratamiento relacionadas con el VIH en África subsahariana. Nuestro objetivo fue identificar la asociación de los síntomas depresivos con los resultados relacionados con el VIH entre una muestra representativa de personas de 18 a 49 años en Sudáfrica. Utilizando modelos de regresión logística (N = 1044), los síntomas depresivos se asociaron inversamente con los que se informaron que habían probado de VIH alguna vez (AOR 0,92, IC del 95%: 0,85 a 0,99; p = 0,04) y la adherencia al TAR (AOR 0,82, IC del 95%: 0,73 a 0,91; p < 0,01) entre las mujeres. Para los hombres, los síntomas depresivos se asociaron positivamente con la vinculación con cuidado (AOR: 1,21, IC del 95%: 1,09–1,34; p < 0,01). La depresión puede tener un impacto adverso en la adherencia al TAR para las mujeres VIH-positivas y reducir la probabilidad de que las mujeres se hagan la prueba del VIH. Para los hombres VIH-positivos, los resultados sugieren que la depresión fomente una conducta de búsqueda de ayuda, afectando así sus interacciones con el sistema de salud. Estos resultados subrayan la necesidad de que los que proveen servicios médicos tengan en cuenta la salud mental en sus programas que abordan los resultados relacionados con la salud.

## Introduction

Depression often provokes experiences of poor behavioral and physical health outcomes in addition to diminished emotional well-being. The co-occurrence of HIV and depression, which is common [1], can exacerbate poor health outcomes in both realms. Depression has been found to be associated with lower uptake of HIV-related care in sub-Saharan African (SSA) settings, including linkage to care, and adherence to antiretroviral therapy (ART) for those who are HIV-positive, and seroconversion [2]. A meta-analysis concluded that the prevalence of major depressive disorder was 13% for HIV-positive adults on ART compared to 24% for HIV-positive individuals not on ART and mixed groups of on, and not on, ART [3]. Other studies within this review using non-diagnostic screening instruments identified depressive symptoms among approximately one third of HIV positive people (range 9 − 32%, by instrument). These figures emerge within a context of under-diagnosed depression, due to a constellation of factors including inadequate mental health training and services, stigmatization of depression, and lack of service integration [3]. Taken together, this likely points to an underestimation of the scope of depression, and its sequelae, in SSA.

South Africa has the highest number of HIV-positive individuals of any country in the world [4]. The majority of studies linking depression to poor behavioral outcomes for HIV-positive individuals in South Africa have been in antenatal care settings, limiting the samples to women of childbearing age [5]. In these examinations, a clear pattern emerges of worse ART adherence and treatment engagement due to depressive symptoms. Other South African studies that have included both men and women, primarily in HIV clinic settings, have found depression to be a significant predictor of disengagement from care [6, 7] as well as failure to obtain viral suppression [8, 9].

While fairly consistent patterns have been found in SSA and South Africa regarding the link between depression and HIV treatment outcomes, less research has examined the impact of depression on HIV-related primary prevention behaviors, such as HIV testing, though there is some indication of reduced prevention uptake in SSA. Most examinations of depression on HIV testing in SSA have been retrospective in nature and only included recently diagnosed HIV-positive participants [10]. In most cases, increased depression was associated with lower likelihood of prior HIV testing, but there is a significant gap with regard to impact of depression on HIV-related behaviors among HIV-negative individuals. Extrapolating from research on accessing health services, depression has been shown to lessen the frequency of seeking health care services [11], which could extend to HIV testing.

Thus, while there is substantial evidence linking depression with adverse HIV-related outcomes, most of the literature from South Africa has come from relatively small clinic-based samples, as opposed to community-based settings. Further, the majority of investigations have been focused on women, or on samples restricted to HIV-positive individuals. We aimed to identify the association between depression and HIV prevention and care engagement by gender, among a large representative sample from a high HIV prevalence, largely rural and understudied area of South Africa. We hypothesized that depression would be inversely associated with HIV testing and care engagement for both sexes.

## Methods

### Study Setting

The current study consists of secondary data analysis of a population-representative survey conducted in Lekwa-Teemane and Greater Taung sub-districts, Dr. Ruth Segomotsi Mompati (RSM) District, North West Province, Republic of South Africa. Dr. RSM district has elevated HIV prevalence, at 20.3% among adults aged 15–49 years. The sub-district populations are rural and largely living in poverty, with employment revolving around the mining industry and agriculture [12]. There is also high rate of unemployment within communities assessed [12].

### Participant Recruitment and Data Collection

The study was designed to be representative of all adults 18–49 years residing within the sub-districts, based on a multi-stage cluster sampling approach. Details of the sampling approach have been described elsewhere [13]. Briefly, Statistics South Africa (StatsSA) selected a sample of enumeration areas (EAs) proportional to size using census data from 2011. We then enumerated all dwelling units (DU) and residents within the twenty-three selected EAs in Lekwa-Teemane and Greater Taung sub-districts. From this data, StatsSA selected a random sample of one adult (18–49 years) per DU in as many as 36 inhabited DUs within each EA (n = 1561 DUs total), including a second individual to be used as a replacement selection if the primary listed individual was determined to be ineligible for participation.

Fieldworkers made up to five separate attempts to locate the sampled individuals. After locating the sampled individuals, fieldworkers confirmed their eligibility and invited those who were eligible to participate. Following written informed consent, surveys were administered by computer-assisted personal interviewing (CAPI) in a private location in the participant’s home in their language of choice [13]. The survey included questions on demographic characteristics, HIV testing history, HIV status, health services utilization, health behaviors, and other individual and community characteristics. Trained community health workers (CHWs) also offered pre-and post-test counseling and point-of-care HIV rapid antibody testing by trained community health workers (CHWs). Participants who tested HIV-positive or who declined HIV rapid testing were asked to provide blood for dried blood spot (DBS) for laboratory HIV diagnosis and viral load testing and offered a study number to call for the results.

Participants were remunerated for their time with an airtime voucher worth approximately $5 USD from a chosen cell phone provider. Participants were referred to local health care facilities for confirmatory HIV testing and CD4 count when testing HIV positive and for sexually transmitted infections (STI) and tuberculosis (TB) services when screening positive. All study procedures were approved by the Committee for Human Research at the University of California, San Francisco; the Human Subjects Division at University of Washington; the Human Sciences Research Council Research Ethics Committee in South Africa; the Policy, Planning, Research, Monitoring and Evaluation Committee for the North West Provincial Department of Health; and the CDC’s Center for Global Health, Human Research Protection. This project was reviewed in accordance with CDC human research protection procedures and was determined to be non-research.

Detailed information on the participant recruitment is presented elsewhere [13]. Briefly, contact was made at 91.7% of DUs and 1,146 individuals were identified as meeting the eligibility criteria. Of these, 1048 (91.4%) consented to participate. Four individuals were incorrectly recruited, resulting 1044 respondents in our analytic sample. Data were collected between January and March of 2014. Rapid HIV testing and/or HIV test via DBS were captured for 71.7% of the sample (n = 745). A total of 218 individuals were identified as HIV positive via testing or self-reported prior positive HIV result. This sample reflects to a total adult population of 92,508 individuals in the two sub-districts, including 15,623 HIV-positive individuals.

### Measures

Socio-demographic characteristics evaluated were self-reported and included: *sex, age group* (18–29, 30–39, 40–49 years), *South African citizen or permanent resident* (yes/no), *employed in the past 12 months* (yes/no), *marital status* (married or living with partner, single or not living with partner, separated or divorced, widowed), *educational attainment* (primary or less, some secondary, completed secondary, completed college/university or technikon), *household food insecurity in the past month* (anyone in household went to bed hungry), *earned income in past month* (yes/no) and *mobility* (the number of months spent away from home in the past year). *Depressive symptomatology* was determined using the Center for Epidemiologic Studies Depression Scale (CESD)-10, which we identified as reliable among men and women in our sample (α = 0.71, respectively), and which had a possible range of 0–30 [14]. Depressive symptoms were assessed for the time period “in the past week”, and organized categorically (low:<10, moderate: 10–14; severe:15–27) [5, 15, 16]. *HIV testing* behaviors were self-reported by participants; we queried whether they had ever been tested and the month and year of their most recent test. Individuals who had tested within the past twelve months were considered to have tested recently. Only individuals reporting negative or unknown HIV status were included in HIV testing analyses, as individuals reporting they had received a positive HIV test were considered ‘known to be HIV positive’. *Linkage to care* was defined and evaluated in two ways: as having seen a provider for HIV-related care ever (linked to care) and seeing a provider and receiving a CD4 test within 3 months of HIV diagnosis (ideal linkage to care). *Retention in care* was defined for those participants clinically designated as ART-eligible, reporting currently being on ART and seeing an HIV care provider every 3 months in the past year; for participants not yet qualifying for ART, retained in care was defined as reporting seeing a care provider and receiving CD4 testing within the past year.

We also asked participants whether they had ever gone for 6 months or longer without HIV-related care after their initial appointment, representing a *lapse in care*. *P*articipants who reported taking at least 90% of their prescribed ART in the past month were defined as *adherent*, and those who reported having had 7 or more days without taking ART in the 12 prior months were classified as having taken an *ART vacation*. This study was conducted prior to the initiation of universal test and treat, which began in 2016.

### Analysis

We calculated proportions and 95% confidence intervals (CIs) to describe overall and sex-specific participant demographic characteristics and HIV testing behaviors. We estimated logistic regression models to assess the relationship between level of depressive symptomatology and HIV testing behaviors by sex among individuals who were not known to be HIV positive (e.g., those who haven’t been tested or previously tested HIV-negative) using weights and survey procedures to account for the multi-stage sample design. We included age group, marital status, educational attainment, past month earned income, and mobility as covariates in adjusted models; we excluded other covariates of interest (e.g., food insecurity) from these models due to collinearity. We followed a similar modeling approach to estimate the relationship between level of depressive symptomatology and linkage to care variables, including *ever linked to care*, *ideal linkage* (linked within 3 months of diagnosis), and *lapse in care* (ever gone 6 or more months without care after initial linkage) and ART adherence variables among those individuals known to be HIV positive. Weights were created using the inverse probability of selection at each stage (EA, DU and person) and adjusted for non-response by EA and replacement DU members to reflect the municipality, age group and sex distributions within the target population [17]. All analyses were performed in Stata 14 (StataCorp, College Station, TX, USA).

#### Sources of Support

This project has been supported by the President’s Emergency Plan for AIDS Relief (PEPFA) through the Centers for Disease Control and Prevention (CDC) under the terms of 5U2GGH000324-02. The findings and conclusions in this report are those of the authors and do not necessarily represent the official position of the funding agencies.

## Results

The sociodemographic characteristics of our sample are presented by gender and self-reported HIV status (known HIV-positive and presumed negative or unknown status), as analytic outcomes vary by whether participants self-reported being HIV-positive (Table 1). Most men with unknown HIV status were younger (53.1% 18–29 years), single but in a relationship (e.g., not married but have a partner) (75.4%), and just over one-third had completed secondary school or higher (35.3%). Known HIV-positive men were slightly older (40.1% age 30–39 years and 39.3% aged 40–49 years), single but in a relationship (62.1%), and one-quarter had completed secondary school or higher (24.9%). Most women of unknown status were younger (51.8% aged 18–29 years), single but in a relationship (68.2%), and 38.1% had completed secondary school or higher. Known HIV-positive women were slightly older (41.6% aged 30–39 and 36.8% aged 40–49), single but in a relationship (57.9%), and 25.5 had completed secondary school or higher. Household food insecurity was lowest among men of unknown status (19.5%) and highest among known HIV-positive women (40.1%).

**Table 1:** Participant Characteristics by Gender and Known HIV Status, North West Province, South Africa

Participant Characteristic	Males					Females			
	Unknown HIV Status		Known HIV Positive			Unknown HIV Status		Known HIV Positive	
	wgt %*	95% CI	wgt %*	95% CI		wgt %*	95% CI	wgt %*	95% CI
Age group									
18–29 Years	53.1	47.2–58.8	20.6	9.1–40.2		51.8	46.7–56.9	21.6	12.7–34.3
30–39 Years	26.2	21.2–31.9	40.1	24.9–57.6		26.6	23.1–30.5	41.6	32.3–51.5
40–49 Years	20.8	16.2–26.2	39.3	22.5–59.0		21.6	17.7–26.0	36.8	26.8–48.1
South African citizen/permanent resident	99.4	98.2–99.8	97.5	82.7–99.7		99.5	97.2–99.1	100.0	-
Employed past 12 months	56.5	49.3–63.5	45.6	27.3–65.2		51.2	44.8–57.6	57.8	45.2–67.5
Marital status									
Married/living with partner	21.3	15.2–28.9	35.0	19.3–54.9		28.6	24.8–32.7	31.8	20.6–45.7
Single/In relationship	75.4	67.6–81.9	62.1	42.8–78.2		68.2	63.8–72.2	57.9	47.1–68.1
Single (separated/divorced)	3.0	1.4–6.1	2.9	0.6–11.8		1.2	0.6–2.3	4.5	0.8–7.4
Single (widowed)	0.0	0.0-1.1	0.0	-		2.2	1.0-4.4	7.8	3.5–16.5
Educational attainment									
Primary or less	20.0	14.6–26.6	38.3	22.8–56.6		19.4	14.0-26.2	28.8	18.5–41.7
Some secondary	44.8	37.1–52.8	36.8	21.6–55.2		42.6	36.3–49.1	45.7	35.7–56.1
Completed secondary	27.7	21.2–35.2	22.4	9.5–44.3		28.8	22.9–35.4	23.2	14.4–35.3
College/University or Technikon	7.6	3.3–16.3	2.5	0.3–17.4		9.3	5.0-16.4	2.3	0.7–7.3
Food insecurity past month	19.5	15.0-24.9	33.8	15.8–58.1		28.6	22.1–36.1	40.1	27.1–54.7
Mean CESD (SD)	6.98 (4.29)		8.21 (6.18)			6.86 (5.11)		7.55 (5.18)	
CESD Category									
Mild Depressive Symptoms	69.4	61.5–76.4	60.4	41.0–77.0		71.2	65.-76.3	70.2	57.1–80.6
Moderate Depressive Symptoms	22.2	17.3–28.1	27.7	13.2–49.0		20.6	16.7–25.0	19.9	11.4–32.6
Severe Depressive Symptoms	8.3	5.5–12.4	12.0	4.5–28.0		8.2	5.4–12.4	9.9	5.1–18.1

*Note: all percentages weighted to account for sampling, non-response, and age/gender of target population*

Mean CESD was 6.98 (SD 4.39) and 6.86 (SD 5.11) for men and women of unknown status, respectively, and 8.21 (SD 6.18) and 7.55 (SD 5.18) for known HIV-positive men and women, respectively (Table 1). For known HIV-positive men, approximately 40% reported either moderate (score of 10–14) or severe (score of 15–27) depressive symptoms; these categories totaled 30% for unknown status men. For both HIV-positive and not known HIV-positive women, approximately 30% reported either moderate or severe depressive symptoms. No significant differences were identified in depressive symptoms and depressive symptom category by known HIV status for men or women (not shown).

### Overall Engagement in HIV testing, Linkage to Care and Retention in Care, Adherence and Retention to ART

Overall engagement in HIV testing by gender is presented in Table 2 [13]. Approximately two-thirds of males with unknown or HIV-negative HIV status had ever tested for HIV, compared to 87% for females. For known HIV-positive individuals, rates of ever being linked to care were over 90% for both males and females. However, rates of ideal linkage to care were lower (62% for males and 55% for females). Among individuals known to be HIV-positive, approximately 68% of males were retained in care, and 77% of females. Reported ART adherence (90% or higher) was 92% of males and 83% of females.

**Table 2:** HIV Testing, Linkage to Care, and ART Adherence by Gender, North West Province, South Africa

	Males		Females	
	%	95% CI	%	95% CI
Individuals reporting negative or unknown HIV status				
HIV Testing				
Ever Tested	66.7	59.3–73.4	87.2	81.4–91.4
Tested, past 12 m	41.0	34.5–47.9	61.6	54.8–68.0
**Individuals known to be HIV positive**				
Linkage to care				
Ever Linked to Care	90.8	80.3–96.0	98.8	96.0-99.6
Ideal Linkage to Care	61.9	40.0-79.8	54.5	39.6–68.7
Lapse in Care (6 Mo + Without Care)	21.6	9.1–43.0	15.7	9.7–24.5
Retained in Care	68.3	45.9–84.6	77.1	67.2–84.7
ART Adherence				
90% Adherent	91.9	75.8–97.6	83.2	70.1–91.3
ART Vacations^a^, past 12 m	2.6	0.3–18.1	4.5	1.8–11.0

*Note: all percentages weighted to account for sampling, non-response, and age/gender of target population. Ideal linkage to care defined as having seen a nurse or doctor for HIV-related care and received CD4 test within 3 months of HIV diagnosis. Retained in care defined as follows - for those participants clinically designated as ART-eligible, retained in care was defined as reporting currently being on ART and seeing an HIV care provider every 3 months in the past year; for participants not yet qualifying for ART, retained in care was defined as reporting seeing a care provider and receiving CD4 testing within the past year*

^*a*^ *having taken 7 or more days ‘off’ of ART in the prior year*

### Effect of Depression on HIV Testing

Level of depressive symptoms was significantly inversely associated with reported ever HIV testing (aOR 0.92, 95% CI 0.85–0.99) among women, but had no impact on recent HIV testing (aOR 0.97, 95% CI 0.92–1.03). No significant relationship between depressive symptoms and ever or recent HIV testing behavior was identified for men (Table 3). Only individuals who had not been tested or previously tested HIV-negative were included in this analysis.

**Table 3:** Relationship between Level of Depressive Symptoms and HIV Testing, Linkage and ART Behaviors by Gender, North West Province, South Africa

	Males							Females					
	**Univariate Models**			**Adjusted Models** ^a^				**Univariate Models**			**Adjusted Models** ^a^		
	**OR**	**95% CI**	**P**	**aOR**	**95% CI**	**P**		**OR**	**95% CI**	**P**	**aOR**	**95% CI**	**P**
**Individuals with unknown HIV status**													
HIV Testing													
Ever Tested	1.00	0.94–1.05	0.913	0.99	0.94–1.05	0.708		0.93	0.86–1.01	0.093	0.92	0.85–0.99	0.037
Tested, past 12 m	1.00	0.95–1.05	0.930	1.00	0.95–1.05	0.978		0.97	0.92–1.03	0.373	0.97	0.92–1.03	0.354
**Individuals known to be HIV positive**													
Linkage to Care													
Ever Linked to Care	1.15	0.93–1.42	0.183	1.21	1.09–1.34	< 0.001		0.92	0.82–1.03	0.135	0.86	0.71–1.05	0.130
Ideal Linkage to Care	1.01	0.86–1.20	0.862	1.14	0.93–1.40	0.215		1.03	0.94–1.13	0.475	1.02	0.91–1.14	0.737
Lapse in Care	0.72	0.50–1.03	0.068	0.68	0.45–1.03	0.071		1.01	0.89–1.15	0.860	1.05	0.89–1.25	0.547
Retained in Care	1.15	0.97–1.37	0.097	1.19	0.99–1.42	0.057		0.99	0.89–1.10	0.889	0.99	0.88–1.11	0.837
ART Adherence^b^													
90% Adherent	1.96	0.84–4.59	0.116	3.31	0.56–19.77	0.182		0.82	0.74–0.91	< 0.001	0.82	0.73–0.91	0.001
ART Vacations^b^, past 12 m	non-estimable			non-estimable				1.09	0.89–1.32	0.396	1.05	0.89–1.23	0.583

*Note: all percentages weighted to account for sampling, non-response, and age/gender of target population. Ideal linkage to care defined as having seen a nurse or doctor for HIV-related care and received CD4 test within 3 months of HIV diagnosis. Lapse in care defined as more than 6 months without care. Retained in care defined as follows - for those participants clinically designated as ART-eligible, retained in care was defined as reporting currently being on ART and seeing an HIV care provider every 3 months in the past year; for participants not yet qualifying for ART, retained in care was defined as reporting seeing a care provider and receiving CD4 testing within the past year*

^*a*^ *adjusted for age group, marital status, educational attainment, prior earning, and mobility*

^*b*^ *adjusted models for adherence outcomes include age group only*

^*c*^ *having taken 7 or more days ‘off’ of ART in the prior year*

### Effect of Depression on Linkage to Care, Retention in Care, Adherence and Retention to ART

Overall engagement in linkage to care and ART adherence by gender is presented in Table 2 [13]. Among individuals who reported being HIV-positive, level of depressive symptoms was not significantly associated with linkage to HIV care among women; however, among men, it was positively associated with ever being linked to care, marginally negatively associated with lapses in care of 6 months or more, and marginally positively associated with retention in care.

For HIV-positive men, each one-unit increase in depressive symptoms was associated with 21% increased odds of having ever linked to HIV care (aOR: 1.21, 95% CI: 1.09–1.34) and 32% reduced odds of having ever gone 6 months or more without having accessed HIV care (aOR: 0.68, 95% CI 0.45–1.03), and a 19% increased odds of having been retained in care (aOR: 1.19, 95% CI 0.99–1.42). We found that level of depressive symptoms was significantly adversely associated with ART adherence for women (aOR 0.82, 95% CI: 0.73–0.91); however, there were no significant effects between depression and adherence among HIV-positive men.

## Discussion

This study investigated whether depressive symptomatology is associated with engagement in care across the HIV treatment spectrum for both men and women in cross-sectional, population-representative sample. We found that depressive symptomatology is associated with engagement in HIV testing and care, although the pattern differs markedly by gender. Among women, depression may both lessen the likelihood of HIV testing for those with unknown or HIV-negative status and adversely impact ART adherence for women who are known HIV-positive. Given the high prevalence of HIV in this setting, any impact on care may carry severe consequences for women either at risk for or coping with HIV. For HIV-positive men, contrary to our hypothesis, depressive symptoms were positively associated with both linkage to care and marginally associated with remaining in care (no lapses in care), suggesting a possible relationship between depressive symptoms and either help-seeking behavior or an increase men’s interactions with the health system.

In the context of HIV, depression has most often been examined as a predictor of ART adherence, while less attention has been placed on how it may impact HIV testing. In one exception, Govender and colleagues reported that increased depression symptoms were significantly associated with lower likelihood of previously testing for HIV among both older and younger women living with HIV in a household-based study in KwaZulu-Natal, South Africa [10]. Similar findings from South Africa were reported by Rane, where severe depression was significantly associated with higher odds of both late testing for HIV and delayed presentation for care (mild and moderate depression had significant but lower effects on both outcomes) [18]. Our findings add to the above studies focused on women living with HIV, suggesting that depressive symptoms play a role in delaying HIV testing for women who do not know their HIV status. This underscores the need for a continued focus on HIV testing efforts for women, especially those women who may not be accessing antenatal (ANC) services, where routine HIV testing is conducted. There appears to be increasing evidence for depressive symptoms needing to be considered as a factor that may impede individuals from learning their status, thereby preventing them from utilizing the appropriate strategies to address their needs regarding HIV prevention or treatment. Though we did not note delayed testing among men in our sample, we propose that improving mental health screening, including in primary care or other general medical clinics, could widely contribute to improved HIV testing outcomes. However, additional research is needed to explore this association for men, who have been absent from many investigations of HIV testing and depression.

Among women, depressive symptoms also suggested a potential negative influence on ART adherence. This finding is aligned with substantial prior evidence, as documented in a meta-analysis by Heestermans and colleagues of predictors of non-adherence for HIV-positive people in SSA (effects for depression, R = 2.54; 95% CI 1.65, 3.91, I^2^ = 52%) [19]. Other studies have documented similar results in South Africa, most often among pre- and post-natal women [5]. We did not find an association between ART adherence and depression for men. Broadly speaking, men in SSA have been found to have worse adherence to ART [19], but less depression, compared to women [20]. Even without a clear association between depressive symptoms and adherence for men in this study, the broader adverse impact of depression warrants efforts to improve mental health treatment. This aligns with recent efforts to integrate mental health care into HIV care in SSA, which has been shown to have promising results in a recent meta-analysis [21–23].

Counter to our predictions, we found a positive association between increased depressive symptoms and linkage to care for men, while no association was found for women. While unexpected, a similar pattern was reported among a sample of men who have sex with men in Cote d’Ivoire, such that increased depression was a significant positive predictor of engagement with sexual health services [24]. One possible explanation for this finding is that men who sought HIV testing and learned their status (and thus linked to care) may be those who were already suffering from symptoms of more advanced HIV, which could also lead to depressive symptoms. It may also be an example of depressive symptoms being a component of help-seeking behaviors more generally [11]. Of note, only 48% of HIV-positive men in our study were aware of their status. Numerous studies have demonstrated that men often waited until they were ill to seek care [25]. Overall, there have been fewer examinations specifically focusing on men and engagement in care, as well as the potential influence of mental health on behaviors and outcomes related to the HIV cascade in the sub-Saharan African context. That said, recent studies focusing on men who have sex with men (MSM) are an exception [26–28]. However, given the high prevalence of depression in South Africa (17% in one large population-based random sample [29]), additional attention is needed to elucidate the impact of depressive symptoms for both men and women on both HIV prevention and treatment outcomes. However, longitudinal studies are crucial in order to determine the direction of effects between depressive symptoms and prevention and treatment outcomes, as most studies are cross-sectional [30].

Some of our findings might also be related to stigma associated with both HIV and depression [31, 32]. Treves-Kagan and colleagues found stigma to be a significant barrier to care for HIV-positive individuals in North West Province, suggesting that improved treatment outcomes do not simply rely on improving access to ART [31]. A recent meta-analysis focused on the association between HIV-related stigma and depression among HIV-positive individuals in South Africa [32] and found that women and young adults may be most impacted, as well as that the relationship between stigma and depression may be bidirectional. However, some of their findings were acknowledged to be limited by the lack of prospective studies. It may also be that our findings regarding the association of depressive symptoms to being less likely to test for HIV (for women) may be a result of anticipated stigma or wanting to avoid being associated with a visibly stigmatized community [33, 34]. Utilizing intervention approaches that come from a common framework, such as the Health Stigma and Discrimination Framework [35] may be better able to improve outcomes adversely influenced by both HIV and depression-related stigma.

The association of depressive symptoms with a range of HIV-related outcomes suggests the need for increased efforts to diagnose and address depression via behavioral or mental health counseling interventions. Evidence for the significant potential of these types of approaches was demonstrated by Safren and colleagues, who completed a trial in Cape Town that successfully improved depression, adherence, and viral suppression for HIV-positive individuals via a nurse-delivered task shifting intervention implemented in Cape Town [36]. Additional protocols are currently underway for similar approaches in South Africa. For example, Fairall and colleagues describe a nurse-led intervention, including mental health components, focusing on reductions in depression along with improved viral load that is currently being implemented in North West Province, South Africa [37]. Other efforts in the field include an intervention in Cape Town, South Africa by Sikkema and colleagues focused on coping skills for HIV-positive women with trauma, aiming to improve rates of treatment engagement via a positive impact on mental health [38]. Additional promising work from Cape Town was reported by Donenberg and colleagues for a family-based pilot trial among adolescent girls and young women which resulted in intervention participants reported significant differences in mental health outcomes including depressive symptoms and anxiety [39]. The described studies from South Africa demonstrate increased efforts to either integrate mental health into care delivery, or improve mental health of individuals with HIV specifically because of the impact of mental health on HIV-related outcomes [40]. These strategies have been examined across other low- and middle-income countries (LMIC), with several meta-analyses offering further support for this approach [21, 41]. Taken together, efforts such as these point to the importance of integrating depression treatment with ART adherence efforts, which may serve to improve HIV-related outcomes such as viral suppression, as well as improving quality of life and mental health outcomes for HIV-positive individuals.

We also found significant findings such that depressive symptoms may be associated with less HIV testing among HIV-negative women. This echoes results linking depression with other HIV primary prevention behaviors such as worse pre-exposure prophylaxis (PrEP) adherence [42]. Collectively, these studies argue for expanding the scope of depression-focused interventions beyond HIV-positive people, as broader health outcomes may be improved by reducing depression and depressive symptoms, including not only HIV-related outcomes, but chronic medical conditions and other health-related behaviors and screenings [43]. Petersen and colleagues described one such effort to incorporate a nurse-delivered general mental health care package into adult primary care settings in North West Province, resulting in an improvement in detection of depression, as well as a significant reduction in depression symptoms following referral to treatment [44]. Future application of this approach will examine the impact on viral suppression and blood pressure [37]. Further, depression has been linked not only to HIV-related behaviors, but also to HIV incidence. Goin and colleagues found that among a large sample (N = 2,415) of adolescent girls and young women, increased depression was associated with subsequent HIV infection, thus emphasizing the need to implement and test mental health-focused interventions and programs which could contribute to reduced numbers of HIV infections [2].

The primary strength of our study stems from our large, population-representative household survey in North West province, South Africa, providing a community setting for examination of these issues outside of the typical clinical setting. However, there are some limitations to this study, including the cross-sectional nature of this analysis and thus the inability to make casual inferences. Additionally, engagement in care outcomes (e.g., linkage, adherence) were based on self-report without verification of clinic records. Thus, reports could be impacted by poor recall of dates or actual attendance and adherence. There may be some survivor bias in that those individuals engaged in care are more likely to survive and have inflated estimates of engagement in care (although findings from this study resemble comparable findings from samples that did have verification of treatment and accounting for participant death). This sample may not reflect a highly mobile population, which is extremely common in the North West Province. Mobile populations may be less likely to be captured in survey data and are more likely to drop out of HIV care. An additional limitation is that data collection for this study was completed prior to the initiation of universal test and treat policies in South Africa, which occurred in 2016 [45]. The initiation of these policies has improved uptake of ART [46], especially for women, but gaps remain for 90-90-90 targets with regard to testing and treatment [47] indicating the need to understand contextual factors that may impede or facilitate uptake of these prevention and treatment approaches.

Taken together, our results suggest that depressive symptoms can exert an influence on HIV-related behaviors in a myriad of ways, and its impact likely varies by gender. These are important points to consider for future programs and research that aim to improve HIV-related behaviors, particularly as there is a burgeoning focus on these issue in sub-Saharan Africa. These findings, from a large representative sample from an area of high HIV prevalence, underscore the need for health care settings to factor mental health conditions, such as depression, more explicitly into their programs, in order to address the entire complement of health-related outcomes, particularly for women.

## Data Availability

Deidentified participant data or other prespecified data will be available subject to a written proposal to the corresponding author and a signed data sharing agreement.

## Code Availability

Code requests will be considered by the study investigators upon written request.
